# SFPQ Promotes Lung Cancer Malignancy *via* Regulation of CD44 v6 Expression

**DOI:** 10.3389/fonc.2022.862250

**Published:** 2022-05-30

**Authors:** Libang Yang, Jianbo Yang, Blake Jacobson, Adam Gilbertsen, Karen Smith, LeeAnn Higgins, Candace Guerrero, Hong Xia, Craig A. Henke, Jizhen Lin

**Affiliations:** ^1^ Department of Medicine, University of Minnesota, Minneapolis, MN, United States; ^2^ Department of Laboratory Medicine and Pathology, School of Medicine, University of Minneapolis, Minneapolis, MN, United States; ^3^ The Cancer Center, Fujian Medical University Union Hospital, Fuzhou, China; ^4^ Hematology, Oncology and Transplantation, School of Medicine, University of Minnesota, Minneapolis, MN, United States; ^5^ Center for Mass Spectrometry and Proteomics, University of Minnesota, St. Paul, MN, United States; ^6^ The Immunotherapy Research Laboratory, Department of Otolaryngology, Cancer Center, University of Minnesota, Minneapolis, MN, United States

**Keywords:** mesenchymal stem cells (MSCs), lung non-small cell (NSC) cancer, nuclear fraction, quantitative proteomics, ingenuity pathway analysis, SFPQ, CD44v6

## Abstract

Mesenchymal stem cells (MSCs) contribute to tumor pathogenesis and elicit antitumor immune responses in tumor microenvironments. Nuclear proteins might be the main players in these processes. In the current study, combining spatial proteomics with ingenuity pathway analysis (IPA) in lung non-small cell (NSC) cancer MSCs, we identify a key nuclear protein regulator, SFPQ (Splicing Factor Proline and Glutamine Rich), which is overexpressed in lung cancer MSCs and functions to promote MSCs proliferation, chemical resistance, and invasion. Mechanistically, the knockdown of SFPQ reduces CD44v6 expression to inhibit lung cancer MSCs stemness, proliferation *in vitro*, and metastasis *in vivo*. The data indicates that SFPQ may be a potential therapeutic target for limiting growth, chemotherapy resistance, and metastasis of lung cancer.

## Introduction

Non-small cell (NSC) lung cancer is one of the most common fatal cancers. Understanding the biological development of NSC lung cancer is critical to improving the treatment efficacy. The progression of lung cancer is dependent on the interaction between tumor cells and the microenvironment composed of different cellular components, including mesenchymal stem cells (MSCs). Due to their various transdifferentiation plasticity, MSCs have recently attracted widespread attention in the development of various diseases and cancers, however, the roles of MSCs in the tumor microenvironment are controversial. They may contribute to tumor growth and elicit anti-tumor immune responses in tumor pathogenesis. The functional mechanisms of MSCs in the microenvironment of NSC lung cancer remain to be clarified (1–5).

We have previously identified intrinsically fibrogenic MSC*s* as the source of IPF fibrosis in the human idiopathic pulmonary fibrosis (IPF) lung and found that the gene expression profile of IPF MSCs is different from MSCs isolated from lung tissue of control patients (6–8). Discovery of genes or proteins in MSCs from NSC lung cancer and how they contribute to lung cancer progression could greatly help in understanding the development of NSC lung cancer and the discovery of novel therapeutic targets.

Spatial proteomics is an evolving powerful technology where the objective is to define the proteome in specific subcellular compartments (9, 10). Quantitative mass spectrometry, combined with interactomics, is a powerful advantage for this purpose (11–17). Abnormalities in nuclear proteins and chromatin organization can alter key cellular processes, lead to cellular dysfunction, and be hallmarks of many diseases (18–20). Our proteomics analysis of MSC nuclear fraction, bioinformatics, and functional analysis with lung cancer MSCs found that SFPQ (Splicing Factor Proline and Glutamine Rich) is the top upstream regulator of lung cancer MSC cell activity when compared with control MPCs. SFPQ has both DNA and RNA-binding domains involved in a variety of cellular activities, including RNA transport, cell cycle regulation, DNA damage and repair, and apoptosis control. Several studies have reported that SFPQ can increase the growth, metastasis, and chemo-resistance of cancer cells such as liver cancer, breast cancer, ovarian cancer, and colorectal cancers, although the precise mechanism by which SFPQ promotes cancer malignant phenotypes remains unknown (21–26).

As a transmembrane receptor for hyaluronic acid (HA) and a co-receptor for many growth factors and cytokines, CD44 is widely overexpressed in a vast array of tumor cells, including cancer stem cells, and is a critical regulator for cell-matrix adhesion, cell growth, EMT, and tumor progression. CD44 frequently shows the heterogeneity of alternative spliced variants (CD44v), which are expressed primarily on stem cells and cancer cells, and is thought to contribute to cancer development and progression (27–31). Among CD44v isoforms, the aberrant expression of CD44v6 has been found in many cancers and is believed to be responsible for cancer progression and metastasis in colorectal cancer, ovarian cancer, prostate cancer, etc (32–39). Our previous studies have shown that CD44 expression in MSCs supports the self-renewal of IPF MSCs. In the current study, we found that CD44v6 expression was reduced when SFPQ was knocked down in lung cancer MSCs. Understanding the relationship between SFPQ and CD44 may help to elucidate the pathological mechanism of NSC lung cancer.

## Result

### Nuclear Protein Profile Analysis Reveals Protein Markers of MSCs From NSC Lung Cancer

**Figure 1:**
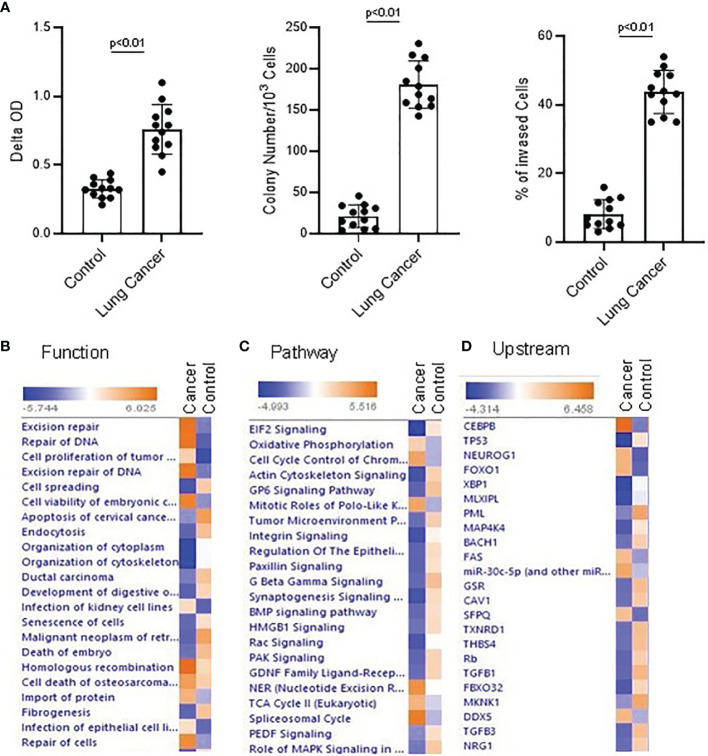
Proteomics and Ingenuity pathway analysis with lung NSC cancer and control MSC nuclear Profile. **(A)** Colony formation, cell proliferation and invasion assay were conducted with the normal and lung cancer MSCs. Lung NSC cancer MSCs have higher capability of proliferation(Left panel), colony forming (middle) and invasion (Right panel) than that of normal MSC cells. 4 normal lung cell lines (C210, C205, C215, C249) and 4 lung NSC cancer cell lines (Can661, Can522, Can838, A549) were used in these experiments. All data are shown as mean ± S.E. (*n*=3 independent experiments). **(B–D)** Proteins identified from control and lung NSC cancer MSC nuclear fraction with relative quantification in Proteomics analysis were applied to IPA to generate the biological networks from Lung cancer MSC and control MSCs dataset. **(B)** Top cell functions associated with the differentially expressed genes. **(C)** Top upstream regulators associated with different proteins. **(D)** Top canonical pathways associated with different proteins. Cell functions, upstream regulators or pathways identified are represented on the y-axis. The x-axis corresponds to the –log of the P-value (Fisher’s exact test) and the orange points on each pathway bar represent the ratio of the number of proteins in a given pathway that meet the cutoff criteria, divided by the total number of proteins that map to that pathway.

In previous studies, we used the cell surface markers CD44 and stage-specific embryonic antigen-4 (SSEA-4) to isolate stem cell-like cells from IPF. It has been shown that CD44+SSEA-4+ double-positive cells preferentially express some stem cell genes (28, 29). Therefore, in the current study, CD44 and SSEA-4 were used as markers for the isolation of MSC cells from the NSC lung cancer and normal lung cells. We found that CD44 and SSEA4 positive MSCs isolated from normal lung cells and NSC lung cancer cells showed the differences in proteomics and ingenuity pathways related to cell stemness, cell proliferation, and invasion (**Figure 1A**). Proteins from the nuclear fraction of those MSCs were then applied to TMT (Tandem Mass Tag) mass spectrometry to be identified and quantified. Global proteomic analyses with MSCs from NSC lung cancer and control group identified and quantified 6,015 proteins, which present in the nuclear fraction of all cell groups. Between these cell groups, 1,576 proteins (26% of the total protein) were observed to be significantly different (**Supplementary Table 1** **).**

When using Ingenuity Pathway Analysis to analyze these nuclear protein data, there are significant differences between lung cancer MSCs and normal control MSCs in terms of cell function, upstream regulatory factors, and signal transduction pathways. Following a published differentiation protocol, the proteomics data was applied to IPA and signal transduction pathway was analyzed with IPA. A review of active cell functions in cancer and normal MSCs indicated the most active are cell DNA damage, cell differentiation, and proliferation, and cell movement. Many proteins were expressed differently and were involved in different functions. For example, ASCC3, POLR2A, CBX8, SMURF2, AQR, PARP, etc. were related with cell DNA damage. UBE2M, C1QBP, CAT, TNC, ACTN4, RNF40, EGFR, CLIC4, etc. were related with cell differentiation. AK4, PFN1, PIP4K2C, RAC1, EGFR, etc. were related with cell movement and migration. In IPA analysis, DNA repair and cell proliferation are higher in cancer MSCs than in normal controls, while cell apoptosis was lower than controls (**Figure 1B**; **Supplementary Table 1**). In canonical pathway analysis, the most active pathways in NSC lung cancer-MSCs were oxidative phosphorylation, cell cycle control, and EIF2 signaling pathways (**Figure 1C**; **Supplementary Table 2**). CEBPB, TP53, FOXO1, SFPQ, etc. are top upstream regulators, which are more dominant in NSC lung cancer-MSCs than controls (**Supplementary Table 2**) (**Figure 1D**; **Supplementary Table 3**). When we review the details of those regulators, they are all relative to cancer development (40–46), and SFPQ plays a role in a variety of biological processes related to cancer progression.

### SFPQ Is Highly Expressed in NSC Lung Cancer-MSCs

**Figure 2:**
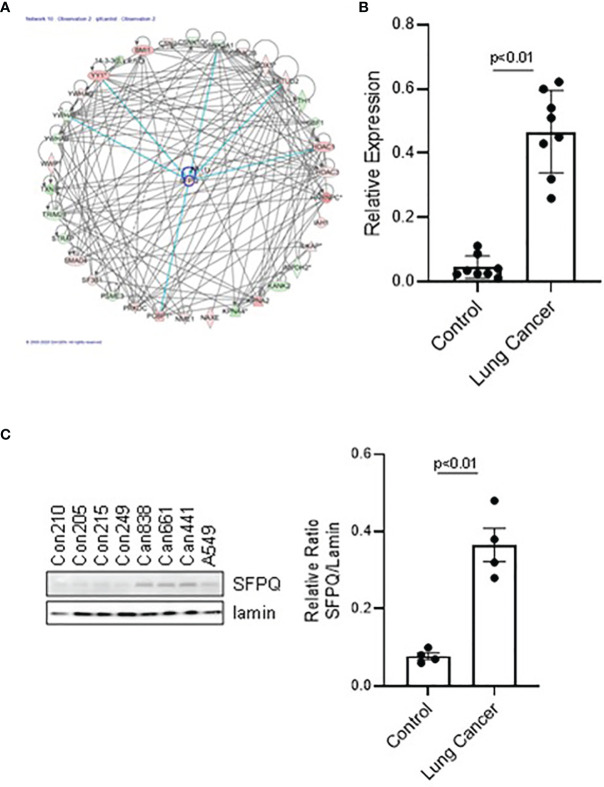
The expression level of SFPQ in lung NSC cancer MSC is higher than that in IPF and control MSC. **(A)** Predicted interactive proteins of SFPQ and their functional interactions are shown by IPA pathway analysis. Primary cell lines were used to measure SFPQ expression level in MSCs. The MSCs were sorted and verified from 4 normal lung cell lines (Con210, Con205, Con215, Con249) and 4 lung NSC cancer cell lines, (Can661, Can522, Can838 and A549) as described in method. **(B, C)** SFPQ expression was analyzed with **(B)** RT-PCR in mRNA and **(C)** western blot analysis in protein levels. Densitometry values were shown in the right graph. All data are shown as mean ± S.E. (*n*=3 independent experiments).

Our proteomics and IPA results showed that the SFPQ level in lung cancer-MSC is well distinguished from the controls and is at the top of upstream regulators. SFPQ is an important protein that maintains the function of stem cells throughout the development process and plays a role in DNA damage, repair, and the cell cycle regulation (22–24). IIPA found that SFPQ interacts with many important proteins (**Figure 2A**), such as YY1, RTN4, RICTOR, HDACs, BMI1, and HNRNPC, which are important in the development of cancer (44, 47–50). When we examined SFPQ expression in NSC lung cancer-MSCs and control MSCs, we confirmed that the expression of SFPQ in mRNA and protein level was significantly higher in NSC lung cancer-MSCs than the controls by RT-PCR and western blot analysis (**Figures 2B, C**), indicating the SFPQ may be an important potential functional biomarker for NSC lung cancer.

### SFPQ Knocking Down Reduces the Abnormal Phenotypes of Cell Stemness, Proliferation, Chemo-Resistance, and Invasion in NSC Lung Cancer-MSC Cells

**Figure 3:**
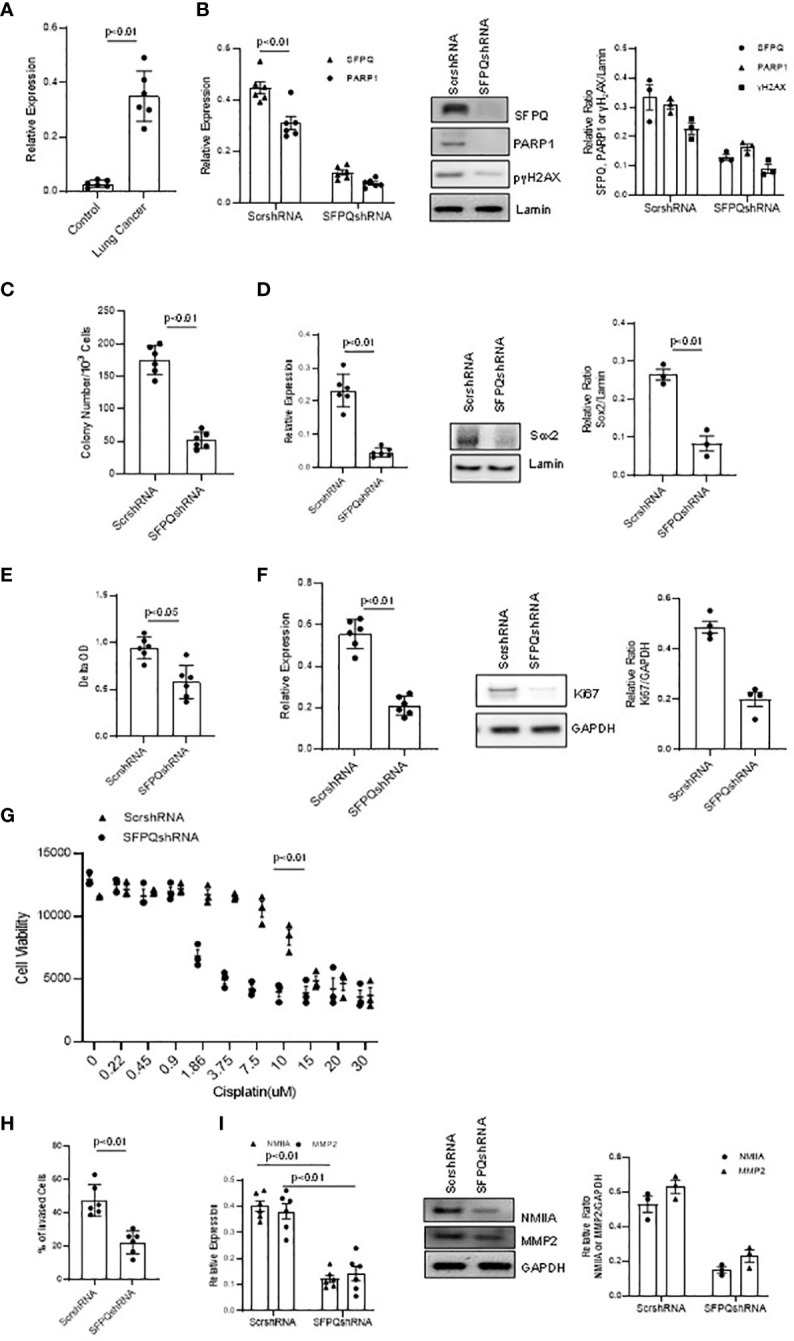
SFPQ is essential for cell stemness, proliferation, and invasion of lung NSC cancer MSCs. Lung NSC cancer MSCs isolated from A549 and Can661 cell lines were transduced with scramble or SFPQ shRNA and 48 hours later the cells were used for following analysis. **(A)** PARP1 mRNA level was quantitatively analyzed with RT-PCR in control and lung NSC cancer MSCs (far left: cell Con210, Con205, Con249; NSC cancer cell lines, Can661, Can838 and A549). **(B)** DNA damage marker H2AX was reduced in PARP1 knockdown lung cancer MSCs. γH_2_AX and PARP1 mRNA level in RT-PCR (left) and protein levels were analyzed with western blot analysis (middle). Densitometry analysis of WB were shown in the right graph. **(C)** Colony Formation Assay of lung cancer MSCs. Colony number was accounted microscopically from 6 random fields/well. Colony number was reduced in lung cancer MSCs transduced with SFPQ shRNA. **(D)** Sox2 expression in lung NSC cancer MSCs was quantified with RT-PCR (left panel) and western Blot analysis (middle panel). Densitometry analysis of WB are shown in the right graph. **(E)** Cell Proliferation assay. Lung cancer MSC proliferation was inhibited when SFPQ expression was knocked down with SFPQ shRNA. **(F)** Ki67 levels in lung cancer MSCs were analyzed with RT-PCR (Left) and western blot analysis (Middle). Densitometry values are shown in the right graph. **(G)** IC50 assay for Cisplatin. Dose responses of cisplatin were plotted as the percent of MTS staining vs. untreated cells from three replicate experiments. Lung NSC Cancer MSCs transduced with SFPQ shRNA were more sensitive to Cisplatin than the control group. **(H)** Cell invasion assay. Bars represent the total number of invading cells from 6 random fields/well. Invasive capacity of lung NSC cancer MSCs was decreased after SFPQ knockdown. **(I)** NMIIA and MMP2 expression in lung NSC cancer MSCs were quantified with RT-PCR (Left) and western blot analysis (Middle). Densitometry values are shown in the right hand graph. All data are shown as mean ± S.E. (*n*=3 independent experiments).

SFPQ was previously reported to be involved with DNA repair (21, 23). In order to determine if SFPQ affects DNA damage and repair in NSC lung cancer -MSCs, we knocked down SFPQ with SFPQ shRNA, and then we measured the levels of DNA repair marker PARP1 and DNA damage marker γ2HAX. We found that the expression of PARP1 was higher in lung cancer–MSCs, and the SFPQ knocking down reduced the levels of PARP1 and γ2HAX in NSC lung cancer -MSCs (**Figures 3A, B**). These results imply that SFPQ is an important regulator in DNA damage and repair.

SFPQ was reportedly involved in the maintenance of cell stemness (23). We then observed the effect of SFPQ on the expression of stemness marker Sox2 and colony-forming ability in lung cancer-MSCs. When knocked down SFPQ with SFPQ shRNA in lung cancer MSCs, the number of colonies was reduced and the expression of stemness marker Sox2 was inhibited in mRNA and protein levels by RT-PCR and western blot analysis (**Figures 3C, D**). These suggest that SFPQ regulates stemness and self-renewal in lung cancer-MSCs.

SFPQ is also related to cancer cell proliferation (26). When comparing the proliferation rate between the lung cancer-MSCs and the control group, the cell growth of lung cancer-MSCs was 38% higher than that of the controls. Ki67 staining with cultured MSCs showed that knocking down SFPQ reduces lung cancer-MSC proliferation and Ki67 expression (**Figures 3E, F**). We also measured cytotoxicity of the Cisplastin in those lung cancer-MSCs and results showed that MSCs with knocking down SFPQ were more sensitive to Cisplastin and IC50 dropped from 9.0 µM in scramble shRNA-transduced cells to 1.9 µM in SFPQ shRNA-transduced MSCs (**Figure 3G**). These results suggest that SFPQ affects cancer MSCs proliferation and resistance to cancer chemotherapy.

Several studies have indicated that SFPQ is involved in cancer cell invasion and metastasis (22, 51). In our invasion assay, the invaded cell rate of cancer MSCs transduced with scramble shRNA was much higher than that of SFPQ knockdown MSCs (**Figure 3H**). NMIIA and MMP2 are considered as a cell migration marker and invasion marker, respectively. RT-PCR and western blot analysis were performed on the expression of NMIIA and MMP2 in MSCs. The results showed that the expression levels of MMP2 and NMIIA were significantly reduced in SFPQ knockdown NSC lung cancer-MSCs (**Figure 3I**). These demonstrate that loss of SFPQ expression significantly decreased the invasive phenotype of NSC lung cancer-MSCs.

### SFPQ Promotes the Malignant Phenotypes of NSC Lung Cancer-MSCs *via* Regulating CD44v6 Expression

**Figure 4:**
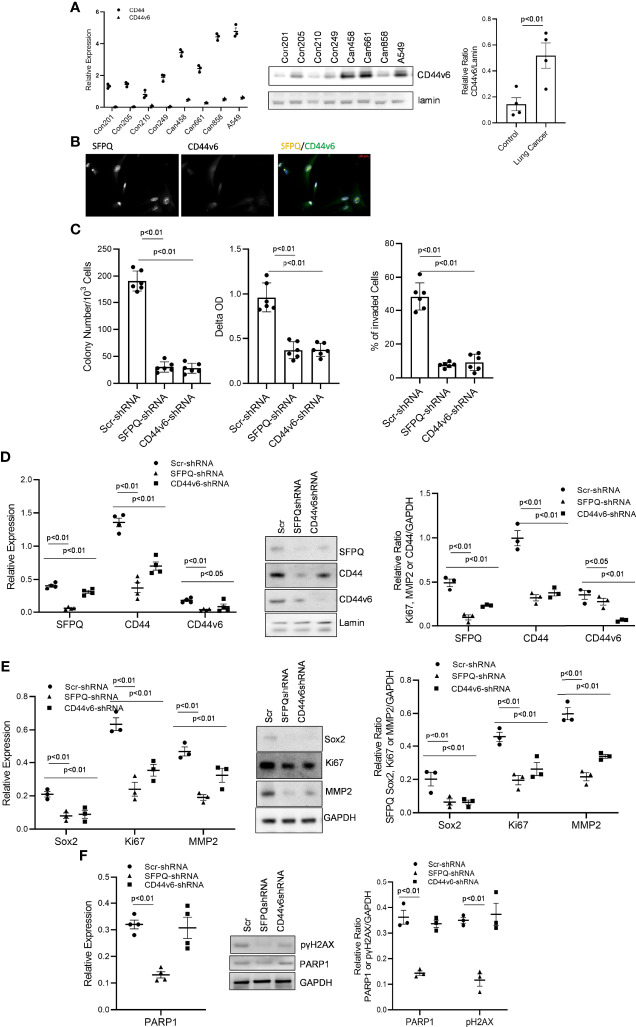
SFPQ promotes the malignant phenotype of lung NSC cancer MSCs *via* regulating CD44v6 expression. **(A)** Control and lung NSC cancer MSCs isolated from 4 normal lung cell lines (Con210, Con205, Con215, Con249) and 4 lung NSC cancer cell lines (Can661, Can522, Can838 and A549) were used to evaluate the CD44v6 levels, Which were quantified with RT-PCR (left, control vs IPF: CD44 p<0.01; CD44v6 p<0.05) and western blot analysis (middle). Densitometry analysis of WB was shown in the right graph. **(B)** localizations of SFPQ and CD44v6 were analyzed by the confocal microscopy with anti-SFPQ (Abcam, USA) and anti-CD44v6 (Abcam, USA) in lung NSC cancer MSCs. CD44v6 is located in both cytoplasm and nucleus. Scale Bar=20µm. **(C–E)** Lung NSC cancer MSCs isolated from A549 and Can 661 cell lines were transduced with scramble or SFPQ shRNA or CD44v6 shRNA Lenti virus for 48 hours, and the cells were used for following analysis: **(C)** Colony Assay (left), Cell proliferation assay (middle) and Cell invasion assay (right). Colony number, cell proliferation rate and invaded cells in lung NSC cancer MSCs transduced with SFPQ shRNA or CD44v6 shRNA were reduced when compared with the control group. **(D)** SFPQ, CD44, and CD44v6 levels were analyzed by RT-PCR (left) and western blot analysis (middle) on the same cell groups. Densitometry values are shown in the right hand graph. **(E)** Expression levels of Sox2, Ki67 and MMP2 were analyzed with RT-PCR (left) and western blot analysis (middle) on the same cell groups. Densitometry analysis was shown in the graph on the right. **(F)** mRNA level of PARP1 was analyzed with RT-PCR (left), and western blot analysis (middle) of PARP1 protein and phosphorylated γH2AX were conducted on the same cell groups. Densitometry analysis was shown in the right graph. All data are shown as mean ± S.E. (*n*=3 independent experiments).

We further investigated possible mechanisms of SFPQ in lung cancer-MSCs. As a multifunctional nuclear protein and a key splicing factor, SFPQ plays its important roles in RNA splicing. CD44 is one of the proteins affected by RNA splicing, largely observed in cancer cells (27, 52). We first examined if CD44 isoform expression in lung cancer MSC was different from that of normal MSCs. The results showed that CD44v6 was higher in lung cancer MSC cells than that in lung normal cell MSCs (**Figure 4A**). Next, we determined whether the expression of CD44v6 is related to SFPQ. We found the CD44v6 was co-localized with SFPQ in the nucleus of NSC lung cancer-MSCs (**Figure 4B**). Furthermore, CD44v6 expression was reduced in SFPQ knockdown NSC lung cancer-MSCs compared to the control group transduced with scramble shRNA. We then compared the changes in cell function among SFPQ-knockdown, CD44v6-knockdown, and the lung cancer MSCs control group. The number of colonies, cell proliferation rate, and invaded cell number were reduced in lung cancer MSCs with CD44v6 knockdown and SFPQ knockdown compared to the control group transduced with scramble shRNA (**Figure 4C**). The expression levels of related marker Sox2, Ki67, MMP2, and NMIIA were also reduced with the loss of the expression of SFPQ and CD44 v6 (**Figures 4D, E** **)**. When observing the levels of DNA repair marker PARP1 and DNA damage marker γ2HAX in lung NSC cancer MSCs, the SFPQ knock down reduced PARP1 and γ2HAX levels, but CD44v6 knockdown did not affect PARP1 and γ2HAX levels obviously (**Figure 4F**). These results suggest that SFPQ affects colony-forming, cell invasion, and proliferation in NSC lung cancer MSCs *via* regulation of CD44v6 level and has additional mechanisms independent of its impact on CD44v6 for regulating DNA damage and repair.

### SFPQ Is Essential for NSC Lung Cancer-MSCs Distant Metastasis *In Vivo*

**Figure 5:**
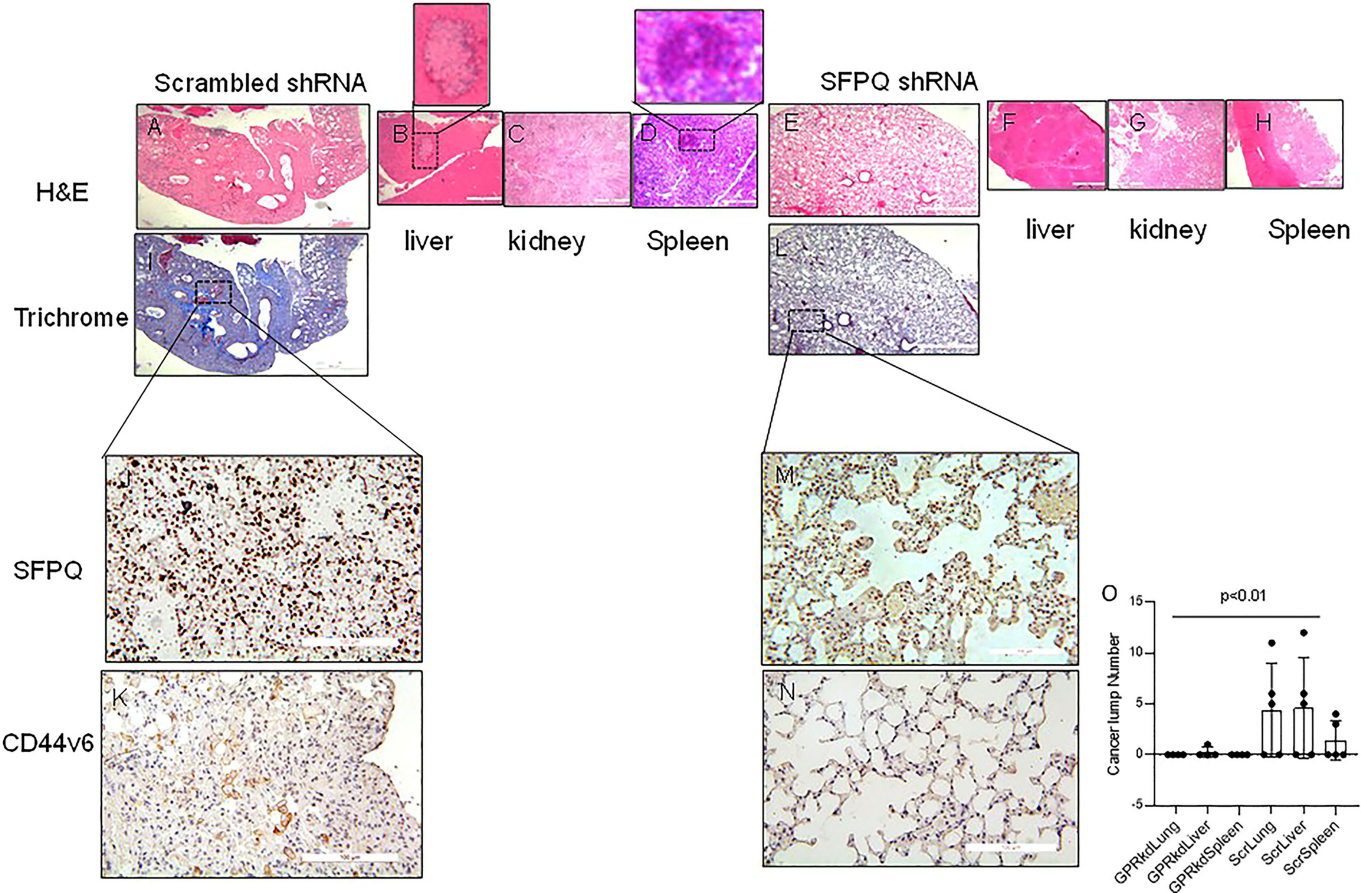
SFPQ is essential for lung NSC cancer MSCs distant metastasis *in vivo*. NSG mice were used for cancer cell metastasis experiment. **(A–N)**. Serial 4 µm sections of the tissues from mice receiving A549 MSCs transduced with scrambled-shRNA **(A–D, I)**, scale bar: 200 µm; **(J, K)** scale bar 50 µm) or SFPQ-shRNA **(E–H, L)**, scale bar: 200 µm; **(M, N)** scale bar 50 µm). Representative H&E and Trichrome staining to assess fibrosis and cancer cell nests. IHC with anti-SFPQ antibody **(J, M)** and anti-CD44v6 antibody **(K, N)** was used to assess the distribution of cells expressing SFPQ and CD44v6 in the lung tissues of mice receiving A549 MSCs transduced with scrambled shRNA or SFPQ shRNA.. **(O)**. The tumor masses in the H&E staining from mouse lung, liver and spleen tissues were counted and summarized in figure O. Compared with mice received SFPQ-knocked down lung NSC cancer MSCs, the mice in the control group had more cancerous masses (Student T test, N=5).

In order to further validate that SFPQ plays a key role in the development of NSC lung cancer, NSC lung cancer-MSCs transduced with SFPQ shRNA and scramble shRNA were intraperitoneal injected (i.p) into the NSG mice, and the different mouse tissues were harvested 6 weeks later to observe the tumor formation and distribution. In mice that received NSC lung cancer-MSCs transduced with scramble shRNA, tumor lumps were presented in the lung (3/5), liver (3/5), and spleen (2/5), but no tumors were observed in mice that received SFPQ shRNA-transduced NSC lung cancer MSCs (**Figure 5O**). Consistent with this result, in tissue IHC analysis of mice that received the control cancer MSCs, large areas of metastatic cancer cells were present in the lung, liver, and spleen tissues (**Figures 5A–D, I**). In contrast, fewer metastatic tumors were observed in the lung, liver, and spleen tissues in mice that received SFPQ-knockdown lung cancer MSCs (**Figures 5E–H, L**). IHC analysis further demonstrated that there were a large number of human CD44v6 and SFPQ positive cells in the lung tissues of mice receiving the control NSC lung cancer-MSCs (**Figures 5J, K**), while there were no cells expressing human CD44v6 in the lung tissues of mice receiving SFPQ knockdown MSCs (**Figures 5M, N**), indicating SFPQ plays an important role in cancer metastasis and SFPQ knockdown could block metastasis. Together, this data indicates that SFPQ plays a key role in promoting the metastasis of NSC lung cancer-MSCs *in vivo*.

## Discussion

Understanding the biology of cancers is critical to improving the treatment of lung cancer. MSCs appear as a key player in tumor pathogenesis by contributing in tumor microenvironments, tumor growth, and eliciting antitumor immune responses (4, 53–56). Screening the different proteins between the normal and cancer MSCs could find key players which are responsible for cancer initiation and development (9, 10). Spatial proteomics and TMT are the most powerful proteomics methods to identify and quantify the hallmarks of many diseases including cancer (57, 58). Reviewing our proteomics and IPA results, we notice there are some proteins translocated abnormally into the nucleus in NSC lung cancer-MSCs compared to the normal control MSCs. Many of them (such as CD44, thioredoxins) are known as cytoplasm, plasma membrane proteins, or extracellular proteins. In the IPA results of NSC lung cancer-MSCs, it was found that some highly expressed nuclear proteins such as POLR2A, CBX8, SMURF2, AQR, PARP, TNC, ACTN4, RNF40, EGFR, CLIC4, are related with DNA damage, cell proliferation, apoptosis, and migration, which are the characteristics of cancer stem cells. These nuclear proteins could be resources for further studies on their relationship with cancer stem cells. Our proteomic results reveal that CEBPB, TP53, FOXO1, SFPQ, etc. are the top upstream regulators, which are more dominated in NSC lung cancer-MSCs than the control group and there are many other proteins such as PML, BACH1 and CEBPB, etc that have not been investigated well in lung cancer. Those proteins are good candidates for further functional studies of NSC lung cancer-MSCs.

SFPQ is an important protein in the maintenance of stem cell development and is also related to cancer proliferation and metastasis (21–26). Our results suggest that SFPQ is critical in the stemness, proliferation, chemoresistance, and cell invasion of lung cancer MSCs. Studies in other laboratories have reported that SFPQ depletion reduces the proliferation of colorectal cancer cells and melanoma cells and induces S phase arrest in the cell cycle. In epithelial ovarian cancer cells, the SFPQ/SRSF2 pathway has been shown to play a key role in regulating chemotherapy-induced apoptosis (24). These results are consistent with ours, indicating that SFPQ may play a similar function in lung NSC cancer-MSCs, but the detailed mechanism of SFPQ on lung cancer cell proliferation, chemoresistance, and metastasis needs further studies.

Since SFPQ plays a key role in RNA splicing, which is important for protein processing (24), we assume it might promote cancer progression *via* regulating RNA splicing. CD44 is a protein involved in cancer initiation and development and highly affected by RNA splicing, thus we hypothesized that CD44 splicing were regulated by abnormal expression of SFPQ in lung cancer. Among CD44v isoforms, CD44v6 plays a major role in cancer progression. The aberrant expression of CD44v6 has been found in many cancers such as colorectal cancer, ovarian cancer, and prostate cancer, and is an independent negative prognostic marker (32–36, 59). In breast cancer, *via* binding growth factors produced by tumor microenvironment, CD44v6 through MAPK pathway promotes cancer cell migration and invasion (32, 34, 60–62). Our results suggest SFPQ affects CD44 isoform v6 level to regulate cell stemness, cell invasion, and cell proliferation, but the change of CD44v6 expression does not affect DNA damage and repair, therefore SFPQ may affect other proteins in those biological processes. How SFPQ affects cancer cell stemness and its upstream regulators are unclear, and mechanism works among SFPQ, CD44v6, and other proteins in lung cancer MSCs need be further investigated.

SFPQ has been reported responsible for metastasis in colorectal cancer and nasopharyngeal carcinoma (22, 51). *In vivo* study, knockdown of SFPQ in NSC lung cancer-MSCs reduces their ability to metastasize distantly, which suggests that SFPQ is a potential therapeutic target for cancer metastasis, although clear mechanisms need to be described. In summary, our data shows that SFPQ not only regulates cancer cell proliferation, stemness, chemoresistance, invasion, and metastasis, but also serves as an upstream regulator of CD44v6. Therefore, it could be a powerful therapeutic target for lung NSC cancer.

## Materials and Methods

### Cell Cultures and FACS Sorting

Primary cells for NSC lung cancer and control cases were harvested from the lung tissue biopsy of adult donors according to a protocol approved by the University of Minnesota Institutional Review Board. Culture supplies were obtained from Thermal Scientific except where noted. MSCs were enriched, purified, and cultured as described previously (28, 63, 64). For isolation of MSCs, primary mesenchymal cells were labeled with mouse anti-human SSEA4 antibody conjugated to Alexa Fluor^®^ 647 (Clone MC-813-70; Catalogue #560796; BD Biosciences, Franklin Lake, NJ) and mouse anti-human CD44 conjugated to FITC (Clone IM7; Catalogue #103006; BioLegend, San Diego, CA). Cells were sorted on a FACS Aria Cell Sorter (BD Biosciences). Cells with SSEA4+ and CD44+ (relative to mouse IgG3 κ isotype control conjugated to Alexa Fluor^®^ 647, clone J606, catalogue #560803 BD Biosciences and mouse IgM κ isotype control conjugated to FITC, catalogue #402207; BioLegend, respectively) were collected as we previously described (63). For IPF MSC isolation, the FACS Sorter gate was set to collect SSEA4 positive cells at the top 3% of CD44 expression (**Supplementary Figure 1**). The sorted cells were verified with MSC positive markers CD73, CD90, CD105 (R & D System, USA) and negative markers (CD45, CD34, CD11b, CD79a, HLA-DR).

### Mesenchymal Progenitor Cells Culture

Cell suspensions of MSCs were maintained in MSC SFM CTS (Thermo Scientific/Gibco, Rochford IL, USA) (37°C, 5% CO_2_). Medium is changed every day.

### Isolation of Cell Nucleus

Primary MSCs were used to isolate cell nucleus with cell organelles fraction kit (Thermo Scientific, USA) by following manufacturer’s instruction. Nuclear fractions of lung cancer-MSCs and control MSCs were isolated by NE-PER Nuclear and Cytoplasmic Extraction reagents (Thermo Scientific, USA).

### Strong Cation Exchange (SCX) Chromatography, LC-MALDI and 4800 MS/MS, and Peptide and Protein Identification

Peptide/protein isolation and identification were conducted as described previously (65, 66). Protein concentrations were determined in desalted samples with Bradford reagent (Bio-Rad, Hercules, CA) and samples containing equal amounts of protein (20 µg) were labeled with MTM reagent (Thermal Scientific, USA) as directed by the manufacturer’s instructions. TMT-based MS was used to obtain proteomes from 6 samples. LC-MS data was acquired for each concatenated fraction using an Easy-nLC 1000 HPLC (Thermo Scientific Inc., Waltham, MA) in tandem with a Thermo Fisher Orbitrap Fusion (Thermo Scientific Inc., Waltham, MA). Peptides were loaded directly onto a 75 cm x 100-µm internal diameter fused silica PicoTip Emitter (New Objective, Woburn, MA) packed in-house with ReproSil-Pur C18-AQ (1.9 µm particle, 120 Å pore; Dr. Maish GmbH Ammerbuch, Germany). The column was heated to 55°C and a flow rate of 300 µL/minute was applied during the gradient. The gradient is as follows: 5-22% Buffer B (A: 0.1% formic acid in water, B: 0.1% formic acid in acetonitrile) for 45 minutes, 22-35% B for 25 minutes, and 35-95% B over 10 minutes. The column was mounted in a nanospray source directly in line with an Orbitrap Fusion mass spectrometer (Thermo Fisher Scientific, USA). Spray voltage was 2.1 kV in positive mode and the heated capillary was maintained at 275°C. The orbital trap was set to acquire survey mass spectra (380–1580 m/z) with a resolution of 60,000 at 100 m/z with automatic gain control (AGC) 1.0E6, 250-ms min injection. EASY-IC was selected for internal mass calibration. The 12 most intense ions (2-7 charged state) from the full scan were selected for fragmentation by higher-energy collisional dissociation with normalized collision energy 35%, detector settings of 60k resolution, AGC 5E4 ions, 250 ms maximum injection time, and FT first mass mode fixed at 110 m/z. Dynamic exclusion was set to 40s with a 10 ppm high and low mass tolerance.

### Database Searching for Protein Identification

The tandem mass spectra were analyzed using Sequest (XCorr Only) in Proteome Discoverer 2.4.0.305 (Thermo Fisher Scientific, Waltham, MA). We used the Uniprot human Universal Proteome (UP000005640) sequence database from July 12, 2019 merged with the common lab contaminant protein database from https://www.thegpm.org/crap/, with a total of 174,234 entries, for the database searching. The Sequest search parameters included: trypsin enzyme, fragment ion mass tolerance of 0.1 Da, precursor ion tolerance 20 ppm, carbamidomethyl cysteine as a fixed modification; pyroglutamic acid from glutamine, deamidation of asparagine, oxidation of methionine, N-terminal protein acetylation, TMT 10plex for lysine, and peptide N-termini as variable modifications.

### Relative Protein Quantification

Scaffold Q+ (v4.9, Proteome Software Inc., Portland, OR) was used for relative quantification of proteins. Peptide identifications were accepted if they could be established at greater than 89.0% probability to achieve an FDR less than 1.0% by the Scaffold Local FDR algorithm. Protein identifications were accepted if they could be established at greater than 5.0% probability to achieve an FDR less than 1.0% and contained at least 2 identified peptides. Protein probabilities were assigned by the Protein Prophet algorithm (67). Proteins that contained similar peptides and could not be differentiated based on MS/MS analysis alone were grouped to satisfy the principles of parsimony. Proteins sharing significant peptide evidence were grouped into clusters. Channels were corrected for incomplete isotope incorporation in all samples according to the algorithm described in i-Tracker (68). Normalization was performed iteratively (across samples and spectra) on intensities, as described in Statistical Analysis of Relative Labeled Mass Spectrometry Data from Complex Samples Using ANOVA (69). Medians were used for averaging. Spectra data were log-transformed, pruned of those matched to multiple proteins, and weighted by an adaptive intensity weighting algorithm. Of 46,922 spectra in the experiment at the given thresholds, 36,422 (78%) were included in quantitation. Differentially expressed proteins were determined by applying Permutation Test with unadjusted significance level p < 0.05 corrected by the Benjamini-Hochberg method.

### Ingenuity Pathway Analysis (IPA)

The lung cancer MSC nuclear proteomic analysis data was imported to the IPA (http://www.ingenuity.com, 2021, May) for functional analysis, canonical pathways, and upstream regulator analysis. Fisher’s exact test was used to calculate a *P*-value, which determines the probability that each biological function and/or disease assigned to the dataset is caused only by chance (70, 71).

### Colony-Forming Efficiency

Single-cell suspension of control and NSC lung cancer -MSCs were incorporated into methylcellulose gels (100ug/ml, Stemcell Technologies, Vancouver, Canada) and maintained in MSC SFM CTS medium (Thermo Scientific/Gibco, Rochford IL, USA) for 1 week at 37°C, 5% CO_2_. Enumeration of colonies was performed microscopically and colony size was quantified by Image J.

### MSCs Proliferation Assay

MSCs proliferation was measured using proliferation kits (Roche, USA). 2X10^4^ single-cell suspension of Scramble or SFPQ shRNA-transduced MSCs were cultured in 96 well plate with MTT reagent for 16 hours and the cells were quantified by following manufacturer’s instruction. Measurements were quantified with a SpectraMax M3 microplate reader (Molecular Devices).

### Invasion Assay

Invasion assay of MSCs was evaluated with the Transwell inserts (8 µm pore) in 24-well tissue culture plates (Millipore, USA). MSCs were cultivated in serum-free DMEM for 24 h, trypsinized and inoculated into the upper chamber at 2X10^4^ cells/well in 300 µl serum-free DMEM. The lower chamber contained 500 µl 10% FBS DMEM (positive control), conditioned DMEM or serum-free DMEM (negative control). After 16 h at 37°C, MSCs were detected with CyQuant GR Dye. Cells remaining in the upper chamber were removed with a release buffer and MSCs that migrated across the insert were quantified with fluorescence reader.

### Cisplatin Resistance Assay

Cisplatin stock was diluted in growth medium to the required concentrations before each experiment. Cells were seeded into 96-well plates at 1.0x1000 cells/well in 100μl of growth media and allowed to adhere overnight. The following day media was removed from wells and replaced with 100µl media containing the indicated treatment or media alone (baseline) in triplicate wells. After 96 hours of treatment, 20µl of MTS reagent (Promega, cat#G3580) was added to each well and incubated in the dark for 2 hours at 37°C, 5% CO2. Absorbance at 570nm was collected on a Bio-Tek 200 plate reader. Each experiment was repeated a minimum of three times.

### Plasmids/Constructs

For loss of function assay, SFPQ was knocked down using shRNA (pGIPZ-SFPQ shRNA; IDT and UMN Genomics center). CD44v6 was knocked down using shRNA (pGIPZ-SFPQ shRNA; Applied Biological Materials Inc. Canada). Scrambled shRNA was served as the control. Cells were transduced using a lentiviral vector containing shRNAs with Polybrene (72).

### Western Blot Analysis

Cells were washed twice in cold PBS and lysed in New RIPA lysis buffer (150 mM NaCl, 50 mMTris pH 8.0, 1 mM EDTA, 1 mM EGTA, 0.5 % sodium deoxycholate, 0.1 % SDS, and 1 % Triton X-100) with protease inhibitor cocktail (0.1 M phenylmethylsulfonyl fluoride, 5 μg/ml leupeptin, 2 μg/ml aprotinin, and 1 μg/ml pepstatin). Protein concentrations of whole cell lysates were determined using the BCA method and equal amounts of each protein sample (15 μg) were separated on an 8~14 % SDS–polyacrylamide gel at 80 V. Separated proteins were then transferred to a polyvinylidene difluoride membrane for 8 minutes on Turbo transfer System (Invitrogen, USA). After blocking with 5 % skim milk powder for 1 h at RT, the membrane was incubated with primary antibody for 1 h at RT or overnight at 4°C. The membrane was washed three times for 15 minutes with 0.05 % PBS-Tween and then incubated for 1 h at RT with the horseradish peroxidase-conjugated secondary antibody. After extensive washing with 0.05 % PBS-T, protein bands were visualized by ECL Plus according to the manufacturer’s instructions (Cell signaling, USA).

### Real-Time Reverse Transcription PCR

Total RNA was extracted with the RNeasy minikit and the cDNA was synthesized with miScript92 RT kit (Qiagen). PCR reactions contained 10 μl SYBR@Green SuperMix (Bio-Rad), 900 nM forward primer, 900 nM reverse primer, and 50 ng cDNA in 20 μl of reaction volume. GAPDH was used as reference, GAPDH was normalized to 1. Reactions were performed in a7900 HT Sequence Detector (Applied Biosystems, USA) with a cycling protocol described before (Applied Biosystems, USA) (73). The primers were listed as follows:

GAPDH Forward: 5′- TGTTGCCATCAATGACCCCTT-3′

GAPDH Reverse: 5′-CTCCACGACGTACTCAGCG-3′

CD44 Forward: 5′-GCTACCAGAGACCAAGACACA-3′

CD44 Reverse: 5′-GCTCCACCTTCTTGACTCC-3′

CD44v6 Forward: 5′-CCAGGCAACTCCTAGTAGTACAACG-3′

CD44v6 Reverse: 5′-CGAATGGGAGTCTTCTTTGGGT-3′

Sox2 Forward: 5′-GGGAAATGGGAGGGGTGAAAAGAGG-3’

Sox2 Reverse: TTGCGTGAGTGTGGATGGGATTGGTG-3’

Ki67 Forward: 5′- TCCTTTGGTGGGCACCTAAGACCTG-3’

Ki67 Reverse: 5′- TGATGGTTGAGGTCGTTCCTTGATG-3’

MMP2 Forward: 5′-CTCAGATCCGTGGTGAGATCT-3′

MMP2 Reverse: 5′-CTTTGGTTCTCCAGCTTCAGG-3′

NMIIA Forward: 5′- AGAGCTCACGTGCCTCAACG-3’

NMIIA Reverse: 5′- TGACCACACAGAACAGGCCTG-3’

SFPQ Forward: 5’-GATCTACAGGGAAAGGCATTGTTG-3’

SFPQ Reverse: 5’-GATACATTGGATTCTTCTGGGCA-3’

RT–PCR products were quantified at the log-linear portion of the curve using LightCycler analysis software and compared to an external calibration standard curve.

### Mouse Xenograft Model of Cancer Metastasis

We utilized NOD/SCID/IL2rγ/B2M (NSG) mouse model to assess the metastatic ability of NSC lung cancer-MSCs *in vivo* (74) Mice were housed under pathogen-free conditions in the University of Minnesota Molecular and Cellular Center Isolation Facility. All mouse studies followed the protocols reviewed and approved by the University of Minnesota Institutional Animal Care and Use Committee (IACUC). An average of 10 weeks of age-matched NSG male and female mice (Jackson Laboratories, USA) were used for intraperitoneal injections for metastasis studies. One million of lung cancer MSCs suspended in 100 μL PBS were IP injected into the mice with a 30-gauge needle after mice were anesthetized with 5% isoflurane. All experimental mice were monitored until fully recovered from anesthesia and were subsequently monitored for disease progression through measuring body weight and behavior signs (pain and distress, et al.) daily. When significant or accelerated losses in body weight (>15%) or mice under distress were detected, mice were euthanized by CO_2_ and different organ tissues were harvested. Histological (H&E and trichrome staining) and immunohistochemical analysis was performed on paraffin embedded mice tissues. IHC using anti-SFPQ antibody (1:500, Ab38148, Abcam, USA) and anti-CD44v6 antibody (1:800,Ab30436, Abcam, USA) to assess the expression of SFPQ and CD44 expressing cells. Specimens were cover-slipped with a Prolong Antifade Kit (Invitrogen/Molecular Probes) and stored overnight at room temperature without light before image analysis.

### Statistical Analysis

All experiments were performed at least in triplicate and results were analyzed using the Student’s t-test or Two-Way ANOVA (for proteomics method described as above). The criterion for significance was P<0.05. Numerical data are reported as means ± standard deviations.

## Data Availability Statement

The dataset has now been deposited to the ProteomeXchange Consortium.via the PRIDE partner proteomics repository, dataset identifier PXD032352.

## Ethics Statement

The studies involving human participants were reviewed and approved by the University of Minnesota Institutional Review Board (University of Minnesota IRB ID: 1504M68341). The patients/ participants provided their written informed consent to participate in this study. The animal study was reviewed and approved by Animal Care and Use Committee (IACUC).

## Author Contributions

LY and JY conceived, designed, and directed the studies with some input from JL and CH. LY and JY wrote the manuscript with assistance from all the authors. LY, BJ, and JY established MSC cell lines, cultured MSCs, performed flow cytometry for isolation of MSCs, performed RT-PCR, Western blot analysis, performed gain and loss of function experiments, and immunohistochemistry. LH and CG: Proteomics analysis. HX and LY: Tissue collection, tissue section preparation and IHC. AG and KS designed and constructed expression constructs and some function study. All authors contributed to the article and approved the submitted version.

## Funding

This work was supported by National Institutes of Health grants R01 HL125227 to CH; R03CA107989 to JL and the Brainstorm Award from the University of Minnesota Cancer Center, 5M Lions International Hearing Foundation to JL.

## Conflict of Interest

The authors declare that the research was conducted in the absence of any commercial or financial relationships that could be construed as a potential conflict of interest.

## Publisher’s Note

All claims expressed in this article are solely those of the authors and do not necessarily represent those of their affiliated organizations, or those of the publisher, the editors and the reviewers. Any product that may be evaluated in this article, or claim that may be made by its manufacturer, is not guaranteed or endorsed by the publisher.
