# Supplementary material for: SFPQ Promotes Lung Cancer Malignancy via Regulation of CD44 v6 Expression
Source: Front Oncol. 2022 May 30;12:862250. doi: 10.3389/fonc.2022.862250 (PMC9190464; doi:10.3389/fonc.2022.862250)
Supplement: Supplementary file 1 [file DataSheet_1.docx]

**Supplementary Figure 1.** FACS Sorting for MSCs. Representative flow cytometry figures showing: A. Gate setting for CD44 and SSEA4 double positive for MSC cells collection; B. Verification of sorted cell with MSC associated antigen CD105 and negative marker cocktail; C. Verification of sorted cell with MSC associated antigen CD90 and negative marker cocktail; D. Verification of sorted cell with MSC associated antigen CD73 and negative marker cocktail.
