# Supplementary material for: SFPQ Promotes Lung Cancer Malignancy via Regulation of CD44 v6 Expression
Source: Front Oncol. 2022 May 30;12:862250. doi: 10.3389/fonc.2022.862250 (PMC9190464; doi:10.3389/fonc.2022.862250)
Supplement: Supplementary file 2 [file Table_1.docx]

**Supplemental Table 1**

**IPA analysis of nuclear protein differences between lung cancer and control MPCs: Top active functions altered between lung cancer and control MPCs**

| Diseases and Bio Functions | Cancer MSC | Control MSC |
| --- | --- | --- |
| Excision repair | 3.287 | 2.845 |
| Repair of DNA | 3.212 | 1.942 |
| Cell proliferation of tumor cell lines | 1.14 | 2.971 |
| Excision repair of DNA | 3.114 | 2.617 |
| Cell spreading | 3.277 | -2.807 |
| Cell viability of embryonic cell lines | 2.866 | 1.456 |
| Apoptosis of cervical cancer cell lines | 1.88 | -1.764 |
| Endocytosis | -2.687 | -1.713 |
| Invasion of cells | 3.01 | 1.745 |
| Organization of cytoskeleton | -4.111 | -1.597 |
| Ductal carcinoma | -2.177 | -2.177 |
| Development of digestive organ tumor | -2.808 | -1.906 |
| Infection of kidney cell lines | 0.85 | -2.121 |
| Senescence of cells | -2.585 | -2.344 |
| Malignant neoplasm of retroperitoneum | -2.677 | -0.786 |
| Death of embryo | -2.405 | -1.665 |
| Homologous recombination | 3.462 | 1.154 |
| Cell death of osteosarcoma cells | 2.288 | 2.098 |
| Import of protein | 1.797 | 2.368 |
| Fibrogenesis | -2.664 | -1.386 |
| Infection of epithelial cell lines | 0.789 | -2.078 |
| Repair of cells | 2.697 | 0.986 |
| Invasion of tumor cell lines | 3.226 | 1.686 |

Quantitative proteomic nuclear protein data was used for ingenuity pathway analysis. Top active functions in control and lung cancer MSCs obtained from Ingenuity pathway analysis with 1576 proteins who are different between lung cancer and controls. The score is generated based on hypergeometric distribution, where the negative logarithm of the significance level is obtained by Fisher's exact test at the right tail.
