# Supplementary material for: SFPQ Promotes Lung Cancer Malignancy via Regulation of CD44 v6 Expression
Source: Front Oncol. 2022 May 30;12:862250. doi: 10.3389/fonc.2022.862250 (PMC9190464; doi:10.3389/fonc.2022.862250)
Supplement: Supplementary file 3 [file Table_2.docx]

**Supplemental Table 2**

**IPA analysis of nuclear protein differences between lung cancer and control MSCs: Top active Canonical Pathways altered between lung cancer and control MSCs**

| Canonical Pathways | Cancer MSC | Control MSC |
| --- | --- | --- |
| EIF2 Signaling | -5.812 | -5.115 |
| Oxidative Phosphorylation | 2.021 | 6.351 |
| Cell Cycle Control of Chromosomal Replication | 3.889 | 3.889 |
| Actin Cytoskeleton Signaling | -4.666 | -2.592 |
| GP6 Signaling Pathway | -3.656 | -2.959 |
| Mitotic Roles of Polo-Like Kinase | 3.873 | 3.357 |
| Tumor Microenvironment Pathway | -3.28 | -3.28 |
| Integrin Signaling | -4.672 | -3.305 |
| Regulation Of The Epithelial Mesenchymal Transition By Growth Factors Pathway | -3.618 | -3.317 |
| Paxillin Signaling | -3.667 | -3.333 |
| G Beta Gamma Signaling | -3.413 | -1.976 |
| Synaptogenesis Signaling Pathway | -4.565 | -1.756 |
| BMP signaling pathway | -3.4 | -3 |
| HMGB1 Signaling | -3.124 | -3.124 |
| Rac Signaling | -4.341 | -3.221 |
| PAK Signaling | -3.55 | -2.197 |
| GDNF Family Ligand-Receptor Interactions | -3.3 | -2.357 |
| NER (Nucleotide Excision Repair, Enhanced Pathway) | 4.715 | 2.774 |
| TCA Cycle II (Eukaryotic) | 3.207 | 3.207 |
| Spliceosomal Cycle | 5.516 | -0.324 |
| PEDF Signaling | -2.6 | -3.4 |
| Role of MAPK Signaling in Promoting the Pathogenesis of Influenza | -3.024 | -1.89 |
| Neurotrophin/TRK Signaling | -3.578 | -2.236 |

Quantitative proteomic nuclear protein data was used for ingenuity pathway analysis. Top active canonical pathways in control and lung cancer MSCs obtained from Ingenuity pathway analysis with 1576 proteins who are different between lung cancer and controls. The score is generated based on hypergeometric distribution, where the negative logarithm of the significance level is obtained by Fisher's exact test at the right tail.
