# Supplementary material for: SFPQ Promotes Lung Cancer Malignancy via Regulation of CD44 v6 Expression
Source: Front Oncol. 2022 May 30;12:862250. doi: 10.3389/fonc.2022.862250 (PMC9190464; doi:10.3389/fonc.2022.862250)
Supplement: Supplementary file 4 [file Table_3.docx]

**Supplemental Table 3**

**IPA analysis of nuclear protein differences between lung cancer and control MSCs: Top upstream regulators altered between lung cancer and control MSCs**

| Upstream Regulators | Cancer MSC | Control MSC |
| --- | --- | --- |
| LARP1 | 6.132 | 2.296 |
| RICTOR | 5.173 | 1.079 |
| CEBPB | 4.962 | 3.077 |
| PPARGC1A | 4.492 | 2.233 |
| let-7a-5p (and other miRNAs w/seed GAGGUAG) | 4.247 | 3.258 |
| aflatoxin B1 | 4.115 | 1.235 |
| valproic acid | 4 | 2.909 |
| EWSR1 | 3.873 | 1.273 |
| rosiglitazone | 3.752 | 3.839 |
| SENP1 | 2.538 | 1.117 |
| Y 27632 | 3.679 | 2.675 |
| TBX2 | 3.668 | 2.909 |
| RABL6 | 3.667 | 2.147 |
| PDPK1 | 2.749 | 1.575 |
| AMPK | 2.725 | 1.643 |
| MITF | 3.598 | 2.679 |
| miR-30c-5p (and other miRNAs w/seed GUAAACA) | 3.566 | 3.131 |
| estrogen receptor | 3.475 | 2.181 |
| MMP3 | 2.655 | 1.138 |
| SFPQ | 3.432 | 0.718 |
| ALDH1A2 | 3.426 | 1.426 |
| INSR | 3.327 | 4.07 |
| Alpha catenin | 3.311 | 1.417 |
| SPDEF | 3.302 | 3.429 |

Quantitative proteomic nuclear protein data was used for ingenuity pathway analysis. Top active canonical pathways in control and lung cancer MSCs obtained from Ingenuity pathway analysis with 1576 proteins who are different between lung cancer and controls. The score is generated based on hypergeometric distribution, where the negative logarithm of the significance level is obtained by Fisher's exact test at the right tail.
